# Spatial variation of pollen receipt and effects of heterospecific pollen on seed set in *Salvia przewalskii*

**DOI:** 10.1002/ece3.9795

**Published:** 2023-02-03

**Authors:** Qiang Fang, Tao Zhang, Benjamin R. Montgomery

**Affiliations:** ^1^ College of Agriculture Henan University of Science and Technology Luoyang China; ^2^ Yunnan Key Laboratory of Plant Reproductive Adaptation and Evolutionary Ecology, Institute of Biodiversity School of Ecology and Environmental Science, Yunnan University Kunming China; ^3^ University of South Carolina Upstate Spartanburg South Carolina USA

**Keywords:** conspecific pollen, heterospecific pollen, interspecific pollen transfer, pollen receipt, seed set, spatial variation

## Abstract

Generalized pollinators visit multiple co‐flowering plant species and may transfer heterospecific pollen grains. Recent studies have indicated that the effect of heterospecific pollen (HP) on reproduction success is variable and depends on the identity of donor and recipient species. However, few studies have documented variation in HP receipt and evaluated the reproductive effects of HP receipt across geographic locations under natural conditions. We investigated the spatial variation of pollen deposition across eight sites and how the pollen receipt related to the seed set of *Salvia przewalskii*, a subalpine perennial herb in Hengduan Mountain in southwest China. We found that stigmatic pollen loads substantially varied among sites for several metrics, including quantities of conspecific and heterospecific pollen, the proportion of HP, and species composition of HP donors. Five different plant families were the most common HP source at one or two sites, and the proportion of HP ranged from 3.4% to 51.3% across sites. The association of conspecific pollen with seed set was positive and variable among sites, whereas the association of HP receipt and seed set was negative and not significantly different among sites. Our results demonstrate variation in the quantity and fitness effect of pollen receipt across sites, which is a precondition for evolution of local adaptation. Further study of variation in patterns and effects of HP receipt for the same recipient species across natural communities would allow better understanding of the ecological and evolutionary consequences of HP receipt.

## Graphical abstract

We documented variations in HP receipt and evaluated its reproductive effects across geographic locations under natural conditions. The association of conspecific pollen with seed production was positive and variable among sites, whereas the association of HP receipt and seed production was negative and not significantly different among sites.

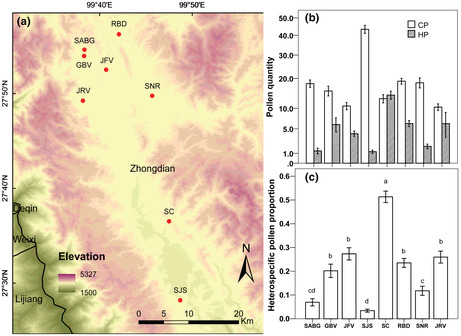

## 1 INTRODUCTION

Studies of plant–pollinator interactions reveal that generalized pollination systems, in which pollinators visit multiple plant species and plants are visited by multiple kinds of pollinators, are common (Waser et al., 1996) and found in a variety of communities (Herrera, 1988; Kaiser‐Bunbury et al., 2017; Olesen & Jordano, 2002). Pollinator movements between plant species can lead to transfer of heterospecific pollen (HP) to plant stigmas (reviewed by Moreira‐Hernández & Muchhala, 2019). HP may affect seed production by multiple mechanisms, including releasing allelochemicals that depress the germination of conspecific pollen (Murphy, & D., Aarssen, Lonnie, W., 1995), occluding the stigmatic surface (Caruso & Alfaro, 2000), or for closely related species by usurping ovules that subsequently abort (Burgess et al., 2008). Effects of HP on plant fitness have been found to vary from being negative to neutral or even positive, with effects depending on the identity of donor species, and with larger heterospecific pollen loads generally causing greater impacts (Ashman & Arceo‐Gómez, 2013; Morales & Traveset, 2008). Moreover, the magnitude of HP's effect on seed production is influenced by the interactions of donor and recipient plant species (Arceo‐Gómez et al., 2019; Lanuza et al., 2021). Negative fitness effects of HP deposition could result in selection of a variety of floral and reproductive traits to reduce pollen sharing (Moreira‐Hernández & Muchhala, 2019). For example, there could be selection to modify flowering phenology to reduce overlap with HP donors (Waser, 1978), to modify where floral parts contact pollinators (Armbruster et al., 2014; Minnaar et al., 2019), or to modify traits to favor reliance on other pollinators (Hopkins & Rausher, 2012). Alternatively, negative fitness effects could select for traits that allow tolerance of HP deposition. For example, larger stigmatic surfaces may provide tolerance to HP because they are less likely to be clogged with pollen (Ashman & Arceo‐Gómez, 2013). However, the effect of selection on traits to avoid HP deposition depends on temporal and geographical variation in patterns and reproductive consequences of HP receipt within a species (Arceo‐Gómez, 2021; Fang et al., 2019). Thus, better understanding of these patterns could be important for understanding HP‐mediated adaptation.

Most studies evaluating the effects of HP have been carried out with potted plants or in greenhouses and have involved experimental manipulation of HP loads, which may lead to an incomplete understanding of natural environmental effects (Ashman & Arceo‐Gómez, 2013; Lanuza et al., 2021). For example, HP receipt could decrease conspecific pollen‐tube growth under resource‐limited but not high‐resource conditions (Celaya et al., 2015). Moreover, in typical HP studies, a certain proportion of HP, ranging from 5% to 20% or higher, from a single‐donor species is applied in an attempt to achieve consistent quantities on all treatment flowers (Caruso & Alfaro, 2000; Da Silva & Sargent, 2011; Moragues & Traveset, 2005; Thomson et al., 1982). In contrast to manipulative experiments, observations of HP incidence under natural pollination regimes have revealed that quantities of HP received tend to be variable and from diverse donor species (Emer et al., 2015; Fang & Huang, 2013; Johnson & Ashman, 2019; Montgomery & Rathcke, 2012; Wei et al., 2021; Zhang et al., 2021). Consequently, effects may differ between manipulative studies and those investigating natural variation (e.g., Montgomery, 2009). Studies that evaluated the impact of natural HP receipt on plant reproduction in the field have observed no detrimental effect of HP (Montgomery, 2009), negative effects on conspecific pollen‐tube success (Parra‐Tabla et al., 2020), or negative interaction between conspecific pollen (CP) and HP quantities, with the effect of CP on seed production becoming weaker with larger quantities of HP deposition (Briggs et al., 2016). Moreover, because the composition of co‐flowering species and pollinators can differ greatly across communities, different populations of the same species may receive HP from different donor species (Herrera, 1988; Johnson & Ashman, 2019), which suggests that the effects of HP receipt may vary among populations across a species' range.

Documentation of patterns of HP receipt across the species’ range would help to understand the importance of pollen transfer in floral evolution and species coexistence (Arceo‐Gómez, 2021; Arceo‐Gómez, Abdala‐Roberts, et al., 2016; Mitchell et al., 2009). A recent study showed consistent patterns of pollen load size and HP proportion in 34 co‐flowering species over three consecutive years, which suggests that propensity for HP receipt is likely a species property rather than the result of occasional random events (Fang et al., 2019). Under such conditions, different strategies to minimize HP effects (avoidance and tolerance) may have evolved. Moreover, if different populations of a plant species were to receive HP from multiple donors in different proportions or if the same HP donor were to differently affect reproductive success among communities, the plant species may experience diverse evolutionary pressures and trajectories. A first step toward understanding HP‐mediated adaptive responses would be to investigate intraspecific variation of HP receipt across multiple communities (Arceo‐Gómez et al., 2019), but few studies have done so. For example, Waites and Ågren (2004) and Arceo‐Gómez, Abdala‐Roberts, et al. (2016) both documented the intensity of HP receipt at multiple sites and found that qualities and compositions of pollen receipt varied significantly among populations. However, the pollen grains were classified into general categories (compatible, incompatible, and heterospecific in Waites & Ågren, 2004; conspecific and heterospecific in Arceo‐Gómez, Abdala‐Roberts, et al., 2016), but not identified to the species level, which limited the interpretation of interspecific pollen transfer. Investigations of variation in HP receipt and its effects on reproductive success across populations of a species could improve understanding of the spatial variation in HP‐mediated selection pressures.

In this study, we collected naturally pollinated stigmas of *Salvia przewalskii* (Lamiaceae) while leaving remaining floral structures undamaged in order to investigate how the pattern of pollen receipt correlated with fruit and seed set. This approach allows linking pollen receipt to seed set within a species across individuals and communities (see Briggs et al., 2016). We identified the HP at the species level and then aggregated counts to the family level, which allowed us to assess interactive effects between pollen from different HP donor families and conspecific pollen on fruit and seed sets. *S*. *przewalskii* is a common subalpine herb in the southwest of China and is known to be pollinated by several bumblebee species (Ye et al., 2017). Frequent interspecific pollinator movements and heterospecific pollen transfer were observed between *S*. *przewalskii* and co‐flowering species (Fang & Huang, 2013, 2016). Our study addresses the following questions: (1) How does pollen composition on *S*. *przewalskii* stigmas vary among sites? (2) How do the quantities of HP from different donors and conspecific pollen affect seed set? (3) Does HP have larger effects at some sites due to being more abundant? (4) Does the relationship between HP receipt and fruit or seed set vary among sites? By addressing these questions, we assess whether there is geographic variation in fitness effects of HP.

## 2 MATERIALS AND METHODS

### 2.1 Study system and sites

*Salvia przewalskii* is a native perennial herb of sub‐alpine meadows, hillsides, or forest margins in Southwest China. Individuals usually produce multiple stems. The branched inflorescence has widely spaced whorls of flowers opening a few a time, with blooming from mid‐July to late‐August. Including side inflorescences, there are up to 60 flowers per individual (Ye et al., 2017). Flowers are zygomorphic and have purple‐red or red‐brown corollas. They are epipetalous with a slightly exserted style and four ovules, which mature into fruits with 0–4 seeds. Flowers typically remain open for 2–3 days.

**FIGURE 1:**
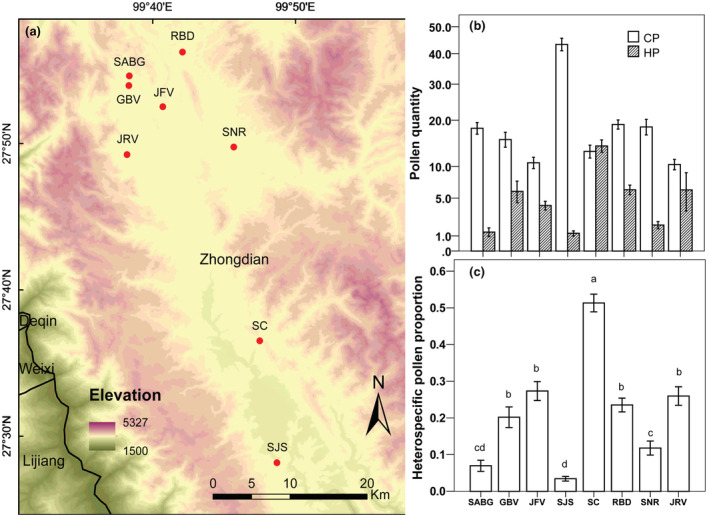
(a) Location of the eight study sites (the coordinates of each location are included in Table S1). (b) Quantities of heterospecific and conspecific pollen deposited on stigmas of each flower at eight sites. (c) The proportion of heterospecific pollen (HP/total grains) at eight sites. Bars represent 95% confidence intervals. Different letters indicate significant differences at *p* < .05.

This study was conducted at eight sites in the southeast of the Hengduan Mountains, Yunnan Province, Southwest China. Study sites were sub‐alpine meadows separated by 2–50 km (Figure 1a, Table S1), and S. *przewalskii* was one of the major flowering species at each site. At each site, one 30 × 30 m plot was established containing about 100–150 flowering *S*. *przewalskii* individuals. Sampling from only one plot per site minimized within‐site variation in the pollination environment. We identified the co‐flowering species in each plot. On average, 15.9 ± 4.2 (mean ± SD) co‐flowering species were in bloom in study plots during our fieldwork.

### 2.2 Stigma and seed collection and pollen identification

Field collections occurred from July 25 to August 19, 2021, during peak flowering of *S*. *przewalskii*. We observed abundant pollinators, suggesting saturated pollination. We labeled 1065 fully blooming flowers (133.1 ± 10.2 per site) from 369 individuals selected haphazardly (Table 1). Density of *S*. *przewalskii* was similar among sites, and it was the most or second‐most abundant flowering species at all sites. We collected 2.9 (median, 3) stigmas per plant, with a range from 1 to 10 stigmas due to variations in plant size. Because the wilted corolla and pistil usually abscise, we secured the corolla to the corona with a single horizontal pin at the corona's midpoint to keep it from falling off. For each site, we performed a given procedure (labeling, stigma collection, or seed counts) for all flowers on the same day (Table S1 for labeling dates). After 3 or 4 days, when the corolla wilted, we removed the stigma with clean forceps and stored it in a microcentrifuge tube containing 70% ethanol. In most cases, the style abscised naturally, which indicated that the fertilization had already occurred, and germinated pollen exines were still affixed to the stigma. Ten days after stigma collection, we counted the developed and undeveloped seeds of each flower, which were readily distinguishable based on size. This approach allowed us to link pollen receipt and seed number in the same flower (Briggs et al., 2016; Waser & Price, 1991). In some cases, stigmas were damaged or flowers (plants) were missing or showed evidence of herbivore damage. In total, 878 stigmas from 335 individuals were retrieved (Table 1). We then observed the stigmatic pollen grains in the laboratory at 400× magnification and captured a series of images using a digital camera (Nikon D90) linked to a microscope (Nikon E100), with one image per species of HP pollen on each stigma, the pollen from each HP species being sufficiently aggregated to be captured in a single image. Pollen grains were identified to the species level on the basis of morphological features including size, shape, and exine ornamentation, with use of a pollen reference library of co‐flowering species within sites (Fang & Huang, 2013, 2016). We excluded the observations for which there was no pollen deposition (10 flowers) or conspecific pollen quantities were smaller than the number of seeds (19 flowers). We assume that these observations were due to loss of stigmatic pollen in the field during fertilization and stigma collection.

**TABLE 1:** The sampling effort, total quantities of conspecific pollen (CP), and heterospecific pollen (HP), as well as species richness of HP donors across all stigmas, richness of co‐flowering community in site's vicinity, and major heterospecific pollen categories for eight study sites in Shangri‐La, Southwest China.

ID	Site	Individuals	Flowers labeled	Stigma and seed counted	Total CP	Total HP	HP species	Community species	Most common			Second‐most common		
									Plant family	% Observed	% Total HP	Plant family	% Observed	% Total HP
1	SABG	52	129	98	1766	128	22	20	Fab	23.5	28.1	Ast	12.2	35.2
2	GBV	40	115	95	1465	564	21	19	Oro	24.2	12.9	Fab	23.2	19.1
3	JFV	34	123	93	992	376	25	17	Ros	28.0	16.0	Ast	23.7	12.2
4	SJS	37	140	117	5068	141	17	8	Lam	14.5	19.9	Oro	11.1	26.2
5	SC	60	143	120	1546	1684	27	18	Ros	74.2	52.1	Ast	36.7	7.1
6	RBD	50	145	114	2158	703	25	16	Oro	71.9	57.6	Ast	22.8	11.1
7	SNR	37	134	104	1907	200	15	11	Ast	24.0	34.5	Oro	11.5	9.5
8	JRV	59	140	108	1118	664	24	18	Ast	38.0	15.2	Oro	24.1	52.1

*Note*: The coordinates of each location are included in Table S1. % Observed, the proportion of stigmas with donor pollen grains from the most or second‐most common donor family. % Total HP, the proportion of all HP pollen from the most or second‐most common donor family.

Abbreviations: Ast, Asteraceae; Fab, Fabaceae; Lam, Lamiaceae; Oro, Orobanchaceae; Ros, Rosaceae.

### 2.3 Data analyses

To test whether *S*. *przewalskii* populations experienced differences in pollen deposition, we compared the quantities of CP and HP received, as well as the proportion of HP across sites. For comparisons of CP and HP among sites, we used GLM (Poisson) with site as a fixed factor, with individual flowers treated as the unit of replication. For comparisons of the proportion of HP among sites, we used a binomial GLM (logit‐link) with the dependent variable consisting of HP (event variable) and total pollen grains (trial variable) and site as a fixed factor (GLM, SPSS 19.0). To test whether *S*. *przewalskii* received HP from similar donor species across sites, we calculated Bray–Curtis dissimilarity of HP compositions between all site pairs. The Bray–Curtis similarity ranges from 0 (no difference) to 1 (completely different HP composition).

The major HP donor species varied substantially across sites (Table 1) and no single species or genus contributed over 30% of total HP. Consequently, we did not test the effect of each HP species or genus individually. Instead, we identified HP to the species level and then summed counts to the family level. For each HP family, one or two species contributed most of the pollen (e.g., *Picris hieracioides* for Asteraceae, *Astragalus pullus* for Fabaceae, *Pedicularis densispica* for Orobanchaceae, and *Potentilla lancinata* for Rosaceae). Even at the family level, most families were too rarely observed to allow for meaningful comparisons of their effects on reproductive success across sites. Consequently, we categorized HP loads into three HP categories based on the observed prevalence of pollen from each HP family: the most common family (Orobanchaceae, Oro hereafter, including 8 species, all in *Pedicularis*, 23.6% observed prevalence and 22.7% contribution of total HP); the second‐most common family (Asteraceae, Ast hereafter, including 10 species, 22.1% prevalence and 11.5% contribution); and “Other,” a category including all 47 species from across 18 families, each family of which individually was found on 19.4% or fewer stigmas (in aggregate, 53.6% prevalence and 65.7% contribution). Table 1 for the major heterospecific pollen categories at each study site.

We examined effects of HP and CP on reproductive success separately for fruit set and seed set. Fruit set was calculated as a binary variable and seed set was calculated per flower, including aborted fruits as zero values for seed set analyses. For analyses of fruit and seed set, we used generalized linear mixed‐effects models with a binomial distribution (GLMMs by maximum likelihood from the R lmerTest package version 4.2.1, Kuznetsova et al., 2017). We treated individual flowers as the unit of replication because models including nesting of flowers within individual plants failed to converge, perhaps because nearly 20% of plants included data from only one flower. For fruit set, analyses focused on the effect of HP quantity but not HP categories, while for seed set, analyses were repeated, first including HP quantity and second instead including HP categories (Oro, Ast, and Other).

The analysis of fruit set and initial analysis of seed set included total HP as an independent variable. For both analyses, we initially selected the best mixed‐effects model (lowest AIC) from candidate models that included fixed factors of CP and HP, site as a random factor, and at least one interaction between variables. One such candidate model included an interaction between CP and HP only; a second model included an interaction between CP and site only; the third model added included interactions between CP and HP as well as between CP and site; and the fourth model followed the third model but added an interaction between HP and site (model 1, Table S2). Comparisons between otherwise identical models with or without interactions between CP quantity and site (CP|Site) and with or without interactions between HP quantity and site (HP|Site) allowed determination of the significance of both interactions. Second, we compared the initial best model from among the four candidate models described above (1) to multiple reduced models, all of which omitted interactions between site and CP or HP (or HP category): (2) a full model including interactions between the fixed factors (CP × HP); (3) additive model (only fixed factors); (4) quantity of conspecific pollen only (CP); and (5) random effects only. The second analysis of seed set followed the same structure but included HP categories (Oro, Ast, and Other) rather than HP quantity. For all analyses, we used Akaike's information criterion corrected for small sample size (AICc) to determine whether the interaction is preserved (Wiqid package in R 4.2.1, Meredith, 2020).

Additionally, we performed a site‐level analysis to investigate the relationship between pollen receipt and seed set. To accomplish this, we calculated pollen receipt averaged across all stigma collections at each site, including average HP receipt, CP receipt, and % HP. We then determined correlations between each of these three pollen measures and seed set per flower by Pearson correlation.

## 3 RESULTS

### 3.1 Pollen receipt

In total, we observed 16,020 conspecific pollen grains and 4460 heterospecific pollen grains (belonging to 58 plant species) from 849 flowers on 335 *S*. *przewalskii* individuals. Conspecific pollen was absent from 10 stigmas (1.2%), and among flowers with conspecific pollen, quantities ranged from 1 to 116 grains (mean ± SD, 18.9 ± 18.8). Heterospecific pollen was observed on 584 stigmas (68.8%), with quantities ranging from 0 to 295 (5.3 ± 13.7). There was significant among‐site variation in the quantities of CP (Wald *χ* ^2^ = 330.21, df = 7, *p* < .01), HP (Wald *χ* ^2^ = 225.26, df = 7, *p* < .01), and the HP proportion (Wald *χ* ^2^ = 2544.99, df = 7, *p* < .01) (Figure 1b,c). Across sites, CP quantities ranged from 10.4 ± 9.9 (site JRV) to 43.3 ± 24.6 (SJS) and HP quantities ranged from 1.2 ± 2.2 (SJS) to 14.0 ± 14.2 (SC) (Figure 1b). The coefficient of variation across sites was larger for HP than CP (*t‐*test, t = 2.89, df = 14, *p* = .01), indicating that the CP quantity was more stable among sites. HP pollen accounted for 21.7% ± 26.1% of pollen deposited across sites (Figure 1c). At site SC, HP contributed over half of pollen deposition (51.3% ± 26.3%), while at site SJS, HP was rarely observed on stigmas (3.4% ± 6.8%). The composition of HP grains varied substantially among sites. The Bray–Curtis dissimilarity of HP compositions between any two sites averaged 0.67 ± 0.09 and ranged from 0.49 to 0.80.

### 3.2 The associations of HP with fruit set and seed set among sites

The associations of CP and HP quantities on fruit set did not significantly differ among sites (Table 2). In all candidate models, the CP quantity was positively associated with fruit set (*p* < .001, Table 3), while the HP quantity had no significant effect (*p* > .18 in all models). Thus, CP quantity but not HP quantity is the primary influence on fruit set.

**TABLE 2:** AIC values and ANOVA comparisons of generalized linear mixed‐effects models using the binomial distribution either with or without interactive random effects of site (CP|Site).

Response variable	Independent variables	Model AIC		Chisq	df	*p* Value
		With CP|Site	Without CP|Site			
Fruit set	CP + HP	985.68	980.19	0.51	3	.917
Seed set (HP)	CP + HP	2123.72	2137.71	20.08	3	<.0001***
Seed set (HP category)	CP + Ast + Oro + Other	2127.36	2140.89	19.64	3	<.0001***

*Note*: Models include data from all sites and were analyzed for the indicated response variables and independent variables of CP (all models) and HP or HP category, as listed parenthetically.

Abbreviations: Ast, Asteraceae pollen; CP, conspecific pollen; HP, heterospecific pollen; Oro, Orobanchaceae pollen; Other, all other HP grains.

* *p* < ·05.

** *p* < ·01.

*** *p* < ·001.

**TABLE 3:** Results of generalized linear mixed‐effects models with binomial distribution analyzed across eight sites for fruit set and number of developed seeds.

(a) Candidate models	K	AICc	AIC delta	AICcWeight
1. Fruit set				
CP + HP	4	980.24		0.450
CP	3	980.69	0.80	0.359
CP + HP + CP|Site	5	982.26	2.02	0.164
2. Developed Seeds (HP)				
CP + HP + CP|Site	7	2123.72		0.998
CP + HP	4	2137.71	13.99	0.001
CP × HP	5	2138.31	14.59	0.001
3. Developed Seeds (HP category)				
CP + Ast + Oro + Other + CP|Site	9	2127.36		0.997
CP + Ast + Oro + Other	6	2140.89	13.53	0.001
CP	3	2141.13	13.77	0.001

(b) Best model	Fixed effects	Coefficient	SE	*p* Value
1. Fruit set				
CP + HP	(Intercept)	0.0049	0.2864	.986
	CP	0.0376	0.0073	<.0001***
	HP	−0.0104	0.0079	.187
2. Developed Seeds (HP)				
CP + HP + CP|Site	(Intercept)	−1.3799	0.2103	<.0001***
	CP	0.0178	0.0034	<.0001***
	HP	−0.0091	0.0045	.043*
3. Developed Seeds (HP category)				
CP + Ast + Oro + Other + CP|Site	(Intercept)	−1.3792	0.2093	<.0001***
	CP	0.0178	0.0034	<.0001***
	Ast	−0.0026	0.0165	.876
	Oro	−0.0058	0.0071	.409
	Other	−0.0116	0.0058	.048*

*Note*: (a) Candidate models and (b) best model for (1) fruit set, (2) developed seeds with predictor variable of HP quantity overall, or (3) developed seeds with predictor variables of HP categorized by pollen family.

Abbreviations: AICcWeight, level of support in favor of the model being the most parsimonious among the candidate model set; Ast, Asteraceae pollen; CP, conspecific pollen; HP, heterospecific pollen; K, total number of estimable parameters; Oro, Orobanchaceae pollen; Other, all other HP grains.

* *p* < ·05.

** *p* < ·01.

*** *p* < ·001.

**FIGURE 2:**
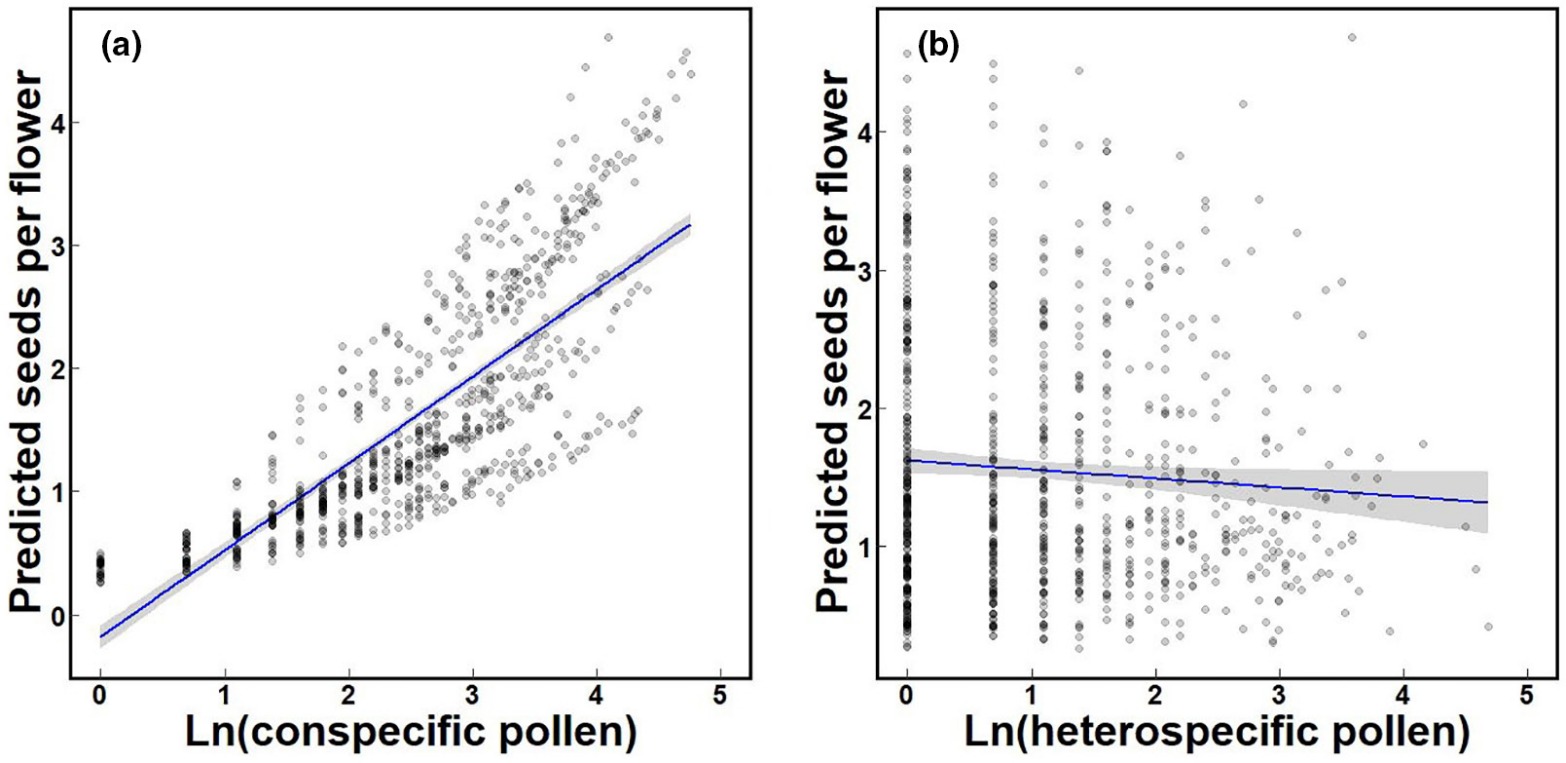
Scatterplots showing relationship between natural log‐transformed conspecific (a) and heterospecific pollen quantities (b) and predicted seed set per flower based on GLMM from Table 3 (b) that best explains developed seeds based on HP quantity. Linear regressions ±95% confidence intervals are depicted.

The association of CP quantity with seed set differed among sites (Table 2). The model that best explained the influence of pollen quantity on seed set included the interaction between CP quantity and site, but not HP quantity and site (ΔAIC = 13.99, Table 3), which indicated that only the interaction with CP quantity significantly improved the model. In this model, there was a positive association of CP quantity (*z* = 5.17, *p* < .001) and negative association of HP quantity (*z* = −2.02, *p* = .043) with seed set (Figure 2). When the HP was separated into three categories, the best model included the interaction between CP quantity and site, but not interactions between any of the HP categories and site or between CP quantity and any HP categories (ΔAIC = 13.53, Table 3). In the best model, CP quantity was positively associated with seed set (*z* = 5.19, *p* < .001); the quantity of Other HP was negatively associated (*z* = −1.98, *p* = .048, Table 3); and neither Asteraceae (Ast) nor Orobanchaceae (Oro) quantities were significantly associated.

**FIGURE 3:**
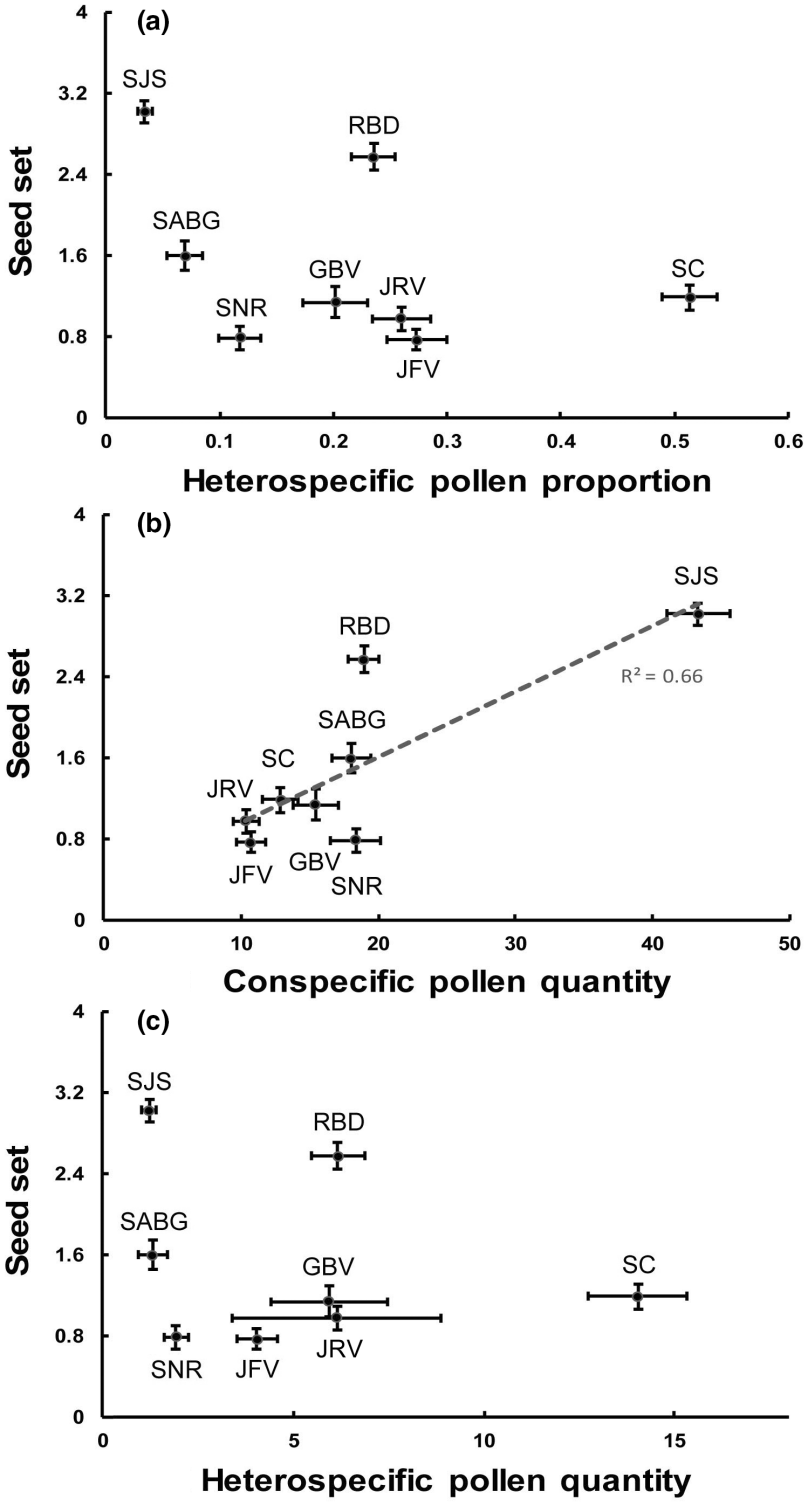
Correlations among HP proportion (a), conspecific pollen quantity (b), heterospecific pollen quantity (c) and seed set per flower across eight sites. Each point represents one site. Bars represent ±SE.

In the site‐level analysis, the amounts of transferred CP and HP varied among sites and correlated with seed set. Flowers on average produced 1.5 ± 0.1 seeds (Mean ± SE). Seed set was highest at SJS, with 3.0 ± 0.1 seeds per flower and lowest at JFV and SNR, with 0.8 ± 0.1 and 0.8 ± 0.1 seeds, respectively (Figure 3). At the level of site, seed set was positively correlated with CP quantity (Pearson correlation, *r* = .82, *p* = .01) but not HP quantity (*r* = −.23, *p* = .58) or HP proportion (*r* = −.40, *p* = .33) (Figure 3). Seed set was not related to the quantity of any pollen category (Figure S1).

## 4 DISCUSSION

In this study, we evaluated variation in HP receipt for *S*. *przewalskii* flowers across eight sites, and the effects of pollen deposition on fruit and seed set under natural conditions. Conspecific pollen receipt was positively associated with fruit and seed set while HP receipt was negatively associated with seed set. The positive association of CP with seed set varied among sites, whereas the negative association of HP with seed set was consistent across sites. However, the quantities of HP and CP, as well as the HP proportions and HP compositions varied substantially across sites, such that the impact of HP on seed set varied among sites. Pollen from Orobanchaceae and Asteraceae, the two most common HP sources, had no measurable detrimental effect on seed set, while the quantity of all other rare HP species was associated with reduced seed set.

Heterospecific pollen was prevalent on stigmas, as has been found in other studies (e.g., Arceo‐Gómez, Abdala‐Roberts, et al., 2016; Fang & Huang, 2013; Wei et al., 2021). In *S*. *przewalskii*, HP grains were deposited on more than two‐thirds of stigmas and represented about one fifth of the total pollen load. So far, there is little empirical indication of the extent of within‐species variation in pollen receipt (see Arceo‐Gómez, 2021). A recent study that investigated pollen receipt in 34 species, including *S*. *przewalskii*, over three consecutive years found temporal stability in patterns of pollen receipt with greater variation in the proportion of HP among species than among years (Fang et al., 2019). Those results suggest that propensity for HP receipt is affected by species traits, not a result of occasional random events. In that study, *S*. *przewalskii* had a comparatively low HP proportion across 3 years, less than 20% (Fang et al., 2019). In the present study, despite substantial variation in CP and HP loads, the proportion of HP was typically low, less than 30% of total pollen receipt at 7 of 8 sites. However, at site SC, the HP and CP quantities were similar, suggesting greater potential for pollen interference to affect reproduction. Further studies of the within‐species variation of pollen receipt could provide better understanding of the cause and consequence of HP deposition (Arceo‐Gómez, 2021; Mitchell et al., 2009).

Under natural conditions, species often receive heterospecific pollen from multiple species at different ratios (Fang & Huang, 2013; Johnson & Ashman, 2019; Wei et al., 2021), but few studies have investigated the effect of heterospecific pollen deposition in the field (Celaya et al., 2015). Similar to Briggs et al. (2016), we found that CP quantity exerts a primary influence on seed set; however, we detected only a direct negative effect of HP on seed set rather than a negative interaction with CP. The effect of HP may depend on interactions between donor and recipient species, with HP having strong detrimental effects on seed set in some donor and recipient combinations, but neutral or positive effects in others (Arceo‐Gómez et al., 2019; Lanuza et al., 2021). In this study, we observed only neutral or negative fitness effects of different HP categories.

Our results demonstrate that the quantity of CP and HP as well as the relationship between CP and seed set vary among sites. *S*. *przewalskii* is self‐compatible but outcrossing increases seed set (Ye et al., 2017). The variable benefit of CP across sites may result from smaller benefits at sites with higher selfing or inbreeding rates. There may be selection for increased floral attractiveness at sites with low CP receipt and traits that promote outcrossing or reduce inbreeding depression at sites with reduced benefits of CP receipt. The variable benefit of CP across sites may also relate to variation in resource limitation—benefits of CP may be reduced at sites where provisions rather than CP receipt limits seed production (Herrera, 1988). The random effect of site was significant in seed set models, which indicated that other factors, such as resource limitation, may cause among‐site variation in seed set (Totland & Birks, 1996). The site habitats were different from meadow to hillside (Table S1). During the observation, we also found that individual size varied among sites. For example, in site SJS, most individuals were large, with five or more stems, allowing collection of an average of 3.8 flowers per plant. By contract, in site SC, most individuals were smaller, with fewer stems, allowing collection of only 2.5 flower per plant. Future work could profitably explore the relationship between resource levels and effects of HP.

We found consistent negative effects of HP but variable HP loads across sites, which suggests that populations may experience different levels of HP interference. Populations that have been continually exposed to a variety of HP donors may experience selection for adaptations that increase tolerance of HP (Kay & Schemske, 2008). For example, smaller HP effects were observed in populations of *Clarkia xantiana* that previously experienced continual exposure to HP compared to populations without such exposure (Arceo‐Gómez, Raguso, & Geber, 2016). However, the potential role of HP receipt as a selection force is largely unknown (Hopkins & Rausher, 2012). Longer‐term study is required to elucidate whether the spatial variation of HP receipt in *S*. *przewalskii* could lead to spatial heterogeneity in selection.

The effect of HP receipt has been typically tested under greenhouse conditions with HP from one or a limited set of donor species manually applied at a constant ratio, often resulting in a substantial decrease in reproductive output (Ashman & Arceo‐Gómez, 2013; Caruso & Alfaro, 2000; Morales & Traveset, 2008). For example, in a recent experimental study with reciprocal interspecific cross‐pollination among 10 species, seed set was significantly reduced in two‐thirds of trials (Lanuza et al., 2021). In hand pollination treatments, the HP proportion is usually different from under natural conditions (Caruso & Alfaro, 2000; Celaya et al., 2015; Da Silva & Sargent, 2011; Lanuza et al., 2021; Moragues & Traveset, 2005), which may artificially inflate fitness effects relative to natural conditions. Nevertheless, we were able to detect deleterious effects of HP even under natural conditions with most flowers receiving small proportions of HP, similar to previous reports that relatively low proportions of heterospecific pollen are associated with reduced seed production under some environmental conditions (Bruckman & Campbell, 2016; Recart et al., 2019). Moreover, a mixture of species contributed HP to *S*. *przewalskii* stigmas, not a single‐donor species as in most hand pollination studies. HP compositions varied substantially among sites due to differences in the co‐flowering species and the tendency of pollinators to transfer pollen on interspecific flights among nearby species (Fang & Huang, 2016; Ye et al., 2020). For example, large quantities of HP from Lamiaceae were observed at SJS but rarely elsewhere. In our system, the richness of HP grains on each stigma varied across sites from 1.4 ± 0.8 to 2.9 ± 1.4 species.

We detected a deleterious effect of HP overall and HP from all sources except the two most common HP families, Asteraceae and Orobanchaceae, neither of which significantly reduced seed set. Statistical constraints may have limited our ability to detect HP effects from these families. Pollen from Asteraceae and Orobanchaceae was only found on 22.1% and 23.6% of stigmas, respectively, while pollen from all other families was found on 53.6% of stigmas, making an effect of pollen from all other families easier to detect. Additionally, pollen from Asteraceae and Orobanchaceae was unevenly distributed across sites, making it harder to statistically disentangle site effects from effects of HP from these families. Alternatively, it is possible that pollen from these families diminishes seed set less than pollen from less common HP sources. In previous studies in the same region, several Orobanchaceae (*Pedicularis*) and Asteraceae (*Picris* and *Aster*) species were prevalent HP donors to a diverse group of recipient species (Fang & Huang, 2013, 2016). There may be more consistent selection to tolerate receipt of HP from frequent donors.

Our results demonstrate variation in the quantity and fitness effect of HP receipt across sites, which is a precondition for local adaptation. Future work should evaluate the spatial–temporal variation in HP receipt and its consequences for seed set. If within‐population HP receipt or the detrimental effect of HP deposition is highly stochastic over years, it would strongly limit the opportunity for HP to influence selection on flower traits. Conversely, if spatial variation in HP receipt and detrimental effects of HP deposition were consistent across years, these conditions would increase the potential for selective effects of heterospecific pollen to influence floral traits. Thus, understanding spatial and temporal variation in patterns of HP receipt and effects would provide insight into selective pressures related to reproductive success of co‐existing plant species. Similarly, observational and experimental approaches to assessing factors that influence HP receipt, such as the co‐flowering species, species arrangement, and pollinator composition, would also help uncover the underlying drivers of selections (Thomson et al., 2019). In conclusion, our study indicated that plant species were exposed to different HP transfer environments across sites. The results of our study highlight the importance of within‐species variation in HP receipt across natural communities. Such studies would help to understand the way that co‐flowering species interact through pollinator sharing and heterospecific pollen transfer, as well as the potential ecological and evolutionary consequences.

## AUTHOR CONTRIBUTIONS

**Qiang Fang:** Conceptualization (equal); data curation (equal); funding acquisition (equal); investigation (equal); methodology (equal); project administration (equal); supervision (equal); writing – original draft (equal); writing – review and editing (equal). **Tao Zhang:** Data curation (equal); formal analysis (equal); investigation (equal); methodology (equal); writing – original draft (equal); writing – review and editing (equal). **Benjamin Montgomery:** Conceptualization (equal); formal analysis (equal); methodology (equal); writing – original draft (equal); writing – review and editing (equal).

## CONFLICT OF INTEREST

The authors declare that they have no confict of interest.

## FUNDING INFORMATION

This work was supported by The National Science Foundation of China (No. 32071535) and Science Foundation of Henan Province (No. 212300410038).

## Supporting information


Figure S1

Tables S1‐S2

## Data Availability

Data are accessible via Dryad; DOI: https://doi.org/10.5061/dryad.z8w9ghxgr
