# Supplementary figures and images for: Spatial variation of pollen receipt and effects of heterospecific pollen on seed set in Salvia przewalskii
Source: Ecol Evol. 2023 Feb 3;13(2):e9795. doi: 10.1002/ece3.9795 (PMC9897956; doi:10.1002/ece3.9795)

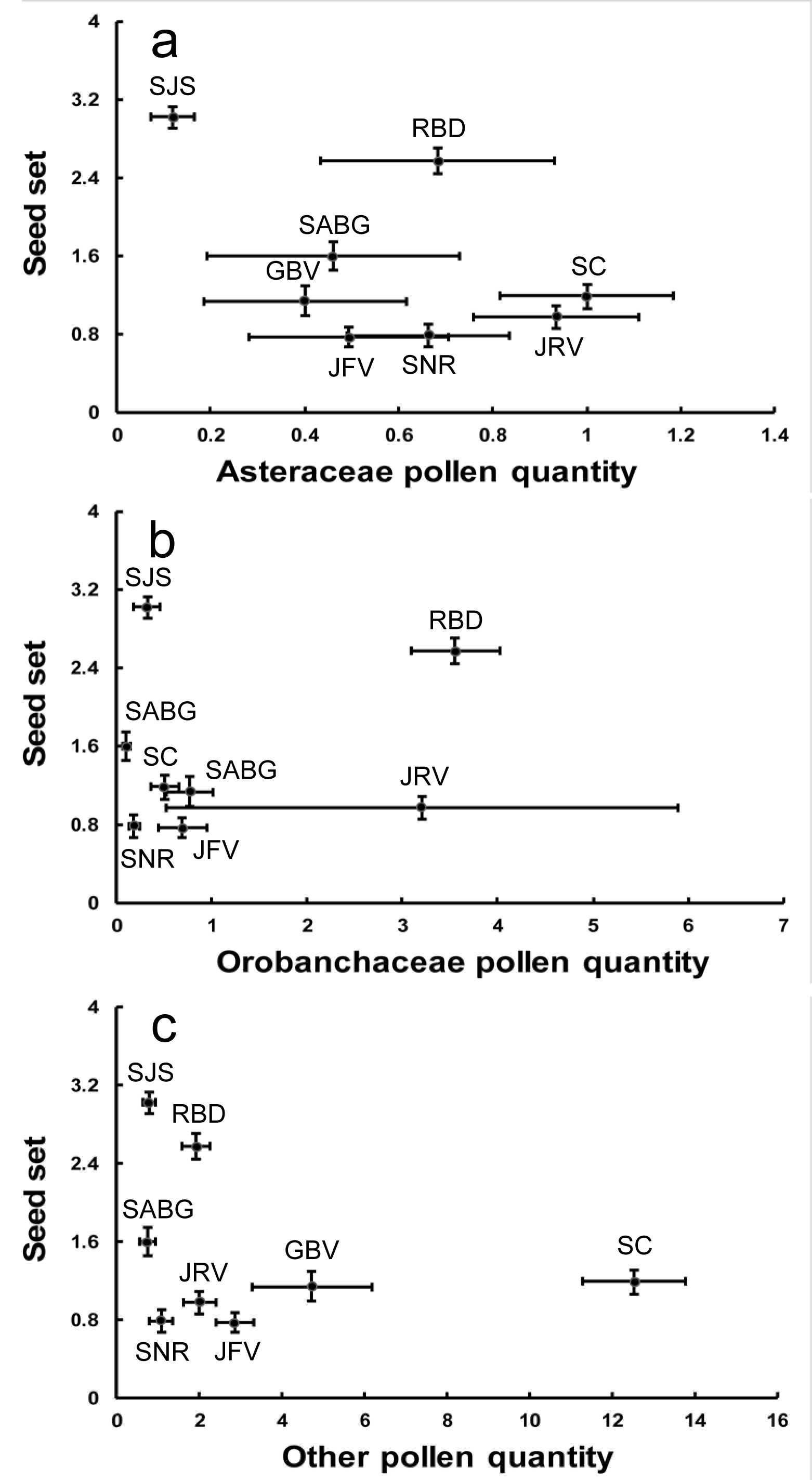

Supplement: Supplementary file 1 — Figure S1 [file ECE3-13-e9795-s001.jpg]
