# Supplementary material for: Spatial variation of pollen receipt and effects of heterospecific pollen on seed set in Salvia przewalskii
Source: Ecol Evol. 2023 Feb 3;13(2):e9795. doi: 10.1002/ece3.9795 (PMC9897956; doi:10.1002/ece3.9795)
Supplement: Supplementary file 2 — Tables S1‐S2 [file ECE3-13-e9795-s002.docx]

Appendix

Appendix Table A1. Location of eight study sites in Shangri-La, Southwest China, as well as the dates of experimental procedures at each site.

| ID | Site | Site name | Habitat | Latitude | Longtitude | Elevation (m) | Date of treatment | | |
| --- | --- | --- | --- | --- | --- | --- | --- | --- | --- |
|  |  |  |  |  |  |  | Mark flower | Collect stigma | Count seed |
| 1 | SABG | Shangri-La Botany Garden | Meadow | 27.9104°N | 99.6393°E | 3345 | 7-25 | 7-29 | 8-8 |
| 2 | GBV | Gongbin Village | Hillside | 27.8993°N | 99.6388°E | 3224 | 7-30 | 8-2 | 8-11 |
| 3 | JFV | Jiefang Village | Hillside | 27.8753°N | 99.6784°E | 3250 | 8-2 | 8-6 | 8-15 |
| 4 | SJS | Shang Jisha | Forest margin | 27.4699°N | 99.8116°E | 3199 | 8-4 | 8-8 | 8-16 |
| 5 | SC | She Chi | Mountain valley | 27.6087°N | 99.7916°E | 3231 | 8-4 | 8-8 | 8-16 |
| 6 | RBD | Re Bida | Hillside | 27.9376°N | 99.7014°E | 3310 | 8-5 | 8-10 | 8-18 |
| 7 | SNR | Sangna Reservoir | Meadow | 27.8294°N | 99.7612°E | 3318 | 8-6 | 8-10 | 8-18 |
| 8 | JRV | Jiaorong Villiage | Hillside | 27.8207°N | 99.6366°E | 3236 | 8-7 | 8-11 | 8-19 |

Appendix Table A2. AIC values and ANOVA comparisons of generalized linear mixed-effects models using the binomial distribution either with or without random effects of Site (CP|Site and HP|Site). Models include data from all sites and were analyzed for the indicated response variables and independent variables of CP (all models) and HP or HP category.

|  | Independent variables | npar | AIC | Chisq | df | p |
| --- | --- | --- | --- | --- | --- | --- |
| Fruit set | CP * HP + (1\|Site) | 5 | 982.2 |  |  |  |
|  | CP + HP + (1\|Site) + (CP\|Site) | 7 | 985.7 | 0.51 | 2 | 0.78 |
|  | CP * HP + (1\|Site) + (CP\|Site) | 8 | 987.7 | 0.02 | 1 | 0.9 |
|  | CP * HP + (1\|Site) + (CP\|Site) + (HP\|Site) | 11 | 992.5 | 1.17 | 3 | 0.76 |
| Seed set | CP * HP + (1\|Site) | 5 | 2138.2 |  |  |  |
|  | CP + HP + (1\|Site) + (CP\|Site) | 7 | 2123.6 | 18.66 | 2 | <0.0001 |
|  | CP * HP + (1\|Site) + (CP\|Site) | 8 | 2125.0 | 0.55 | 1 | 0.46 |
|  | CP * HP + (1\|Site) + (CP\|Site) + (HP\|Site) | 11 | 2131.0 | 0.01 | 3 | 0.99 |
| Seed set (HP category) | CP * HP category + (1\|Site) | 6 | 2140.8 |  |  |  |
|  | CP + HP category + (1\|Site) + (CP\|Site) | 9 | 2127.2 | 19.64 | 3 | <0.0001 |
|  | CP * HP category + (1\|Site) + (CP\|Site) | 12 | 2130.2 | 2.99 | 3 | 0.39 |
|  | CP * HP category + (1\|Site) + (CP\|Site) + (HP category\|Site) | 21 | 2147.3 | 0.89 | 9 | 0.99 |

Appendix Figure A1. Relationships between seed set and pollen quantities of Asteraceae (a), Orobanchaceae (b) and Other HP sources (c) across eight sites. Each point represents one site. Bars represent ± SE.
